# Metagenomic Sequencing for Direct Identification of Candida auris Colonization

**DOI:** 10.1128/mSphere.00638-21

**Published:** 2021-08-04

**Authors:** Teresa R. O’Meara

**Affiliations:** a Department of Microbiology and Immunology, University of Michigan Medical School, Ann Arbor, Michigan, USA

**Keywords:** *Candida auris*, metagenomics, microbiome

## Abstract

Candida auris is an emerging multidrug-resistant yeast that is associated with skin colonization and deadly bloodstream infections, especially in ventilator skilled nursing facilities. An ongoing question is how this organism colonizes the skin of these patients and whether the skin microbiome provides a measure of colonization resistance against C. auris. Now, Huang et al. (X. Huang, R. M. Welsh, C. Deming, D. M. Proctor, et al., mSphere 6:e00287-21, 2021, https://doi.org/10.1128/mSphere.00287-21) demonstrate a method for shotgun metagenomic analysis of the skin to generate a profile of fungal colonization that is highly correlative with culture-based methods. These methods are likely to assist in the diagnosis of C. auris and the identification of microbiome-associated risk factors that predict invasive disease.

## COMMENTARY

Candida auris is an emerging human fungal pathogen that primarily causes hospital outbreaks in immunocompromised patients. One of the defining features of C. auris is its high fungal burden on the skin (1, 2). From the skin, it can then contaminate health care providers, equipment, or other fomite sources, thus providing a reservoir for hospital outbreaks (3). Given the high mortality rates, common antifungal drug resistance, and increased prevalence in hospitals globally, the Centers for Disease Control and Prevention has designated this organism as an urgent public health threat (4). Identification of colonized patients, therefore, is a critical component of preventing spread between patients.

One of the difficulties in identifying C. auris on the skin has been a lack of specificity in diagnostics. A current standard is to use matrix-assisted laser desorption ionization–time of flight mass spectrometry (MALDI-TOF MS) (5) and culture-based methods (6, 7). However, this is labor-intensive, and not all facilities have these assays available, thus leading to an increase in developing PCR-based methods (8, 9). Moreover, patients can be colonized at multiple sites (10), and recent work has demonstrated that the current recommendation for surveillance swabs of bilateral axillae and inguinal creases may not provide sufficient sensitivity for detecting colonization (6, 10). Therefore, increasing the ability to detect C. auris has been an area of intense research focus (11–16).

To increase diagnostic capacity and understand the microbiome features in patients colonized by C. auris, Huang and Welsh et al. (17) developed skin microbiome analysis protocols that can be used directly from surveillance swabs. Their approach used a combination of amplicon and metagenomic analyses to capture multiple aspects of the skin microbiome community. Regular amplicon sequencing of bacterial 16s rRNA gene or fungal ITS1 regions can identify the species that are present in a microbial community, but not the microbial genes that are associated with increased disease, such as genomic variants that are associated with drug resistance. Shotgun metagenomics can allow for analysis of the functional potential of that microbial community, but it is limited in its ability to detect low-abundance members. Together, these sequencing-based approaches can complement culture-based methods in identifying skin microbiome members, especially for those organisms that are difficult to culture (18, 19).

A critical improvement in this technique is the increased resolution of the ITS1 sequences that define C. auris. Previous ITS1 approaches were often only able to resolve organisms to the genus level; here, Huang and Welsh et al. (17) updated their ITS1 databases to be able to differentiate C. auris from other *Candida* species and to identify the clade of C. auris that was present. This clade information is useful, as it helps to demonstrate the clonal outbreak nature of the C. auris colonization in these particular nursing facilities.

First, they tested their sequencing-based approach on confirmed culture-based swabs for C. auris. They were then able to expand and test swabs from two skilled nursing facilities with confirmed C. auris outbreaks and from a comparator facility without C. auris. Their sequencing methods detected the presence of C. auris more often than culturing, but all culture-positive samples were also sequence positive, suggesting that their results are accurate and potentially more sensitive than culture-based methods.

The nonoutbreak skin microbiomes were dominated by *Malassezia*, but in the facilities with C. auris, some samples were nearly 50% C. auris, showing the high levels of colonization that this organism can achieve. Additionally, other *Candida* species were also present on the skin at higher levels, suggesting that colonization by multiple *Candida* species may act as a risk factor for C. auris. Metagenomic analysis of these samples was somewhat limited by low fungal biomass; however, Huang and Welsh et al. (17) were able to examine variants in a few samples with high C. auris colonization. In this set, they were able to confirm clonal expansion, indicative of an outbreak, with only 49 total high-confidence sequence variants among the samples. The underannotated C. auris genome, however, limited their ability to make hypotheses on the functional consequences of these variants.

Skin colonization is generally dominated by bacteria, and in healthy individuals, fungi generally make up a minority of the microbiome (20). Antibiotic treatments are a risk factor for C. auris skin colonization (21, 22), suggesting that colonization resistance by bacteria may play an important role in protecting against C. auris. Therefore, understanding the community composition requires analysis of both kingdoms and how they may interact with each other. Huang and Welsh et al. (17) looked at the members of the bacterial microbiome, and importantly, skin samples that were positive for C. auris were also highly colonized by hospital-associated pathogenic bacteria, including *Proteobacteria*, which is not normally found on healthy adults. This result was recapitulated in the shotgun metagenomics analysis, with community domination by hospital-associated pathogens. This work complements the recent study examining the microbiome associated with C. auris colonization (10).

In summary, Huang and Welsh et al. (17) have developed a robust and accurate method for identifying C. auris in the skin microbiome. These methods will be clinically relevant, as they will increase our power to detect colonization. Although the connections between skin colonization and invasive disease are still under investigation (23, 24), reducing the overall fungal burden in these patients and in these nursing facilities is likely to improve patient outcomes. By including metagenomics in the analysis of the colonizing strain, it is possible that clinicians will be able to identify variants associated with drug resistance or other phenotypes without the need for culturing. Additionally, by including the community structure, it may be possible to associate particular synergies between microbes that are associated with increased disease or pathogenicity. The mechanisms underlying the increased prevalence of hospital-associated bacterial pathogens and C. auris colonization will need to be explored in future work.
